# Long-Cycle Stability of In Situ Ultraviolet Curable Organic/Inorganic Composite Electrolyte for Solid-State Batteries

**DOI:** 10.3390/polym16010055

**Published:** 2023-12-23

**Authors:** Xinghua Liang, Yuying Wang, Zhida Liang, Ge Yan, Lingxiao Lan, Yujiang Wang, Xueli Shi, Shuhong Yun, Meihong Huang

**Affiliations:** 1 Guangxi Key Laboratory of Automobile Components and Vehicle Technology, Guangxi University of Science & Technology, Liuzhou 545006, China; 100001090@gxust.edu.cn (X.L.); wangyy0131@163.com (Y.W.); llx2685062@163.com (L.L.); 13152528815@163.com (Y.W.); s1659186234@163.com (X.S.); 221076946@stdmail.gxust.edu.cn (S.Y.); 2 Guangxi Automobile Group Co., Ltd., Liuzhou 545006, China; 3 School of Automotive Engineering, Guangdong Polytechnic of Industry and Commerce, Guangzhou 510510, China; huangmeih0828@163.com

**Keywords:** Li_4_Ti_5_O_12_, atmospheric plasma spraying (APS), in situ, solid-state batteries, ultraviolet curing (UV-curing)

## Abstract

Lithium-ion solid-state batteries with spinel Li_4_Ti_5_O_12_ (LTO) electrodes have significant advantages, such as stability, long life, and good multiplication performance. In this work, the LTO electrode was obtained by the atmospheric plasma spraying method, and a composite solid electrolyte was prepared by in situ ultraviolet (UV) curing on the LTO electrode. The composite solid electrolyte was designed using a soft–hard combination strategy, and the electrolyte was prepared into a composite of a poly(vinylidene fluoride-co-hexafluoropropylene) (PVDF-HFP) flexible structure and high-conductivity Li_1.3_Al_0.3_Ti_1.7_(PO_4_)_3_ (LATP) hard particles. The composite electrolyte exhibited a good ionic conductivity up to 0.35 mS cm^−1^ at 30 °C and an electrochemical window above 4.0 V. In situ and ex situ electrolytes were assembled into LTO//electrolyte//Li solid-state batteries to investigate their impact on the electrochemical performance of the batteries. As a result, the assembled Li_4_Ti_5_O_12_//in situ electrolytes//Li batteries exhibited excellent rate of performance, and their capacity retention rate was 90% at 0.2 mA/cm^2^ after 300 cycles. This work provides a new method for the fabrication of novel advanced solid-state electrolytes and electrodes for applications in solid-state batteries.

## 1. Introduction

Since the 20th century, lithium-ion batteries have been widely used in smartphones, laptops, and other portable electronic devices. Currently, lithium-ion batteries are also used as the main power source in electric vehicles, which require lithium batteries to have a long cycle life, high rate of performance, safety, reliability, durability, and low cost [1,2,3]. Spinel-structured lithium titanate (Li_4_Ti_5_O_12_, LTO), as a new type of anode material, does not produce lithium dendrites during cycling because of its highly embedded and dislodged lithium potentials (1.55 V vs. Li/Li^+^), and its almost zero volume change during charging and discharging (<0.2%) is why it is known as a zero-strain material, which means that this battery has a long cycle life [4,5,6]. Due to these properties, LTO is used as an anode material with potential applications in electrochemical energy storage, electric vehicles, and grid stabilization.

However, a series of safety issues arising from a thermal runaway caused by the use of liquid electrolytes in lithium-ion batteries have hindered their future development [7,8]. As a result, solid-state batteries are considered the preferred choice for electric vehicles, mobile electronic devices, and other fields. However, solid-state batteries still face many challenges in practical applications, including a poor interfacial contact, high preparation costs, and a relatively low ionic conductivity at room temperature. However, if we can provide a simple and low-cost preparation method to enhance the interfacial contact between the electrolyte and the electrodes, then solid-state batteries will hopefully meet the demand for high-performance solid-state rechargeable batteries to some extent.

Solid polymer electrolytes include poly(ethylene oxide) (PEO) [9,10,11], poly(vinylidene fluoride) PVDF-HFP [12,13], polyacrylonitrile (PAN) [14,15], and the like. Polyvinylidene fluoride-hexafluoropropylene (PVDF-HFP)-based gel polymer electrolytes have been extensively studied due to their electrochemical stability and high dielectric constant. Jie et al. [16] prepared a flexible gel polymer electrolyte based on PVDF-HFP. The ionic conductivity of the gel polymer electrolyte prepared by them is as high as 7.24 × 10^−4^ S cm^−1^, and it exhibits excellent interfacial compatibility with lithium anode at room temperature and high temperatures.

Ceramic electrolytes include garnet-type Li_7_La_3_Zr_2_O_12_ (LLZO) [17] and NASICON-type Li_1+x_Al_x_Ti_2−x_(PO_4_)_3_ (LATP) [18,19,20] or Li_1.5_Al_0.5_Ge_1.5_(PO_4_)_3_ (LAGP) [21]. LATP has attracted great attention due to its wide electrochemical window, high ionic conductivity, and good air stability [22]. However, when the ceramic solid electrolyte is in contact with the metal lithium anode, an electrochemical reaction is prone to occur, which reduces the battery’s performance. A feasible solution is to use ceramic/polymer composite electrolytes. Related studies have shown that the electrical insulation and flexibility of the polymer can not only separate LATP from Li, but also alleviate the interfacial contact resistance [23]. Therefore, the construction of composite electrolytes of LATP and organic polymers (effective combination of LATP and organic polymers) is expected to reduce the interface impedance and further improve the electrochemical performance of LATP. The manufacturing process of the traditional lithium-ion battery cathode is very complicated and causes a certain kind of pollution to the environment. Processes such as sizing and coating require the involvement of toxic organic solvents and subsequent steps such as drying and tableting. The production process of the electrode is complex, the construction period is long, and the production efficiency is low.

The manufacturing process of the traditional lithium-ion battery cathode is very complicated and causes a certain kind of pollution to the environment. Processes such as slurry mixing and coating require the involvement of toxic organic solvents, followed by steps such as drying and tableting. However, the composite electrode prepared by the APS method is more efficient, and the electrode prepared by the APS method does not need to be limited by the size of the coating machine, and it can be used to make a larger macroscopic battery. Moreover, the electrode coating prepared by the APS process has a higher physical strength, and the active material is not easy to peel off from the electrode sheet [24]. Therefore, when the electrolyte is prepared in situ, there will be no separation of the active material from the electrode sheet. In summary, the electrode prepared by the APS process is more suitable for the preparation of in situ batteries, and the in situ electrolyte can effectively reduce the interface impedance caused by the rough electrode surface to the battery, so that the electrode and the electrolyte are in better contact, and the performance of the solid electrolyte is better exerted.

Zhang et al. [25] reported a simple method for interfacial modification using in situ UV-cured gel polymer electrolytes. Compared with other conventional solid-state batteries, solid-state batteries assembled with in situ solid electrolyte membranes exhibit lower interfacial resistance, lower polarization, higher multiplication performance, and more stable cycling performance. Aidoud D. et al. [26] developed a liquid precursor of photoluminescent ionic gels deposited directly onto porous composite electrodes, resulting in an all-solid-state electrode/electrolyte stack upon UV irradiation. Rate performance increased with increasing ionic conductivity, decreased with increasing polymer content, and decreased with increasing oxygen content in the polyacrylate matrix. Shi et al. [27] prepared a thin ZnO layer in situ on both sides of a double-layer Ta-LLZO. Experiments were conducted in order to improve the wettability between the lithium metal and Ta-LLZO (on the anode side), and the results showed that the stability of the battery was improved and the energy density was also improved to a certain extent. Although much progress has been made, the interface problem between the electrolyte and the positive electrode has not been effectively solved. The interface problems of solid-state batteries still hinder the future development of solid-state batteries.

In this work, we provide a new strategy to reduce the interface impedance between solid electrolytes and electrodes and improve the ion conductivity and high electrochemical performance of the electrolytes. LTO electrodes were prepared by atmospheric plasma spraying, and then a polymer/oxide composite electrolyte precursor solution was directly cast onto the surface of the LTO electrode to prepare an electrolyte layer through an in situ UV curing process. The electrolyte layer was assembled with a metal lithium negative electrode to form an LTO//in situ electrolyte//Li solid-state battery. This solid-state battery exhibits better electrochemical performance. This work provides feasible strategies and process methods for preparing flexible, ultra-thin, and large-area solid-state batteries.

## 2. Materials and Methods

### 2.1. Materials

The raw materials included ethoxylated trimethylolpropane triacrylate (ETPTA, Mn = 428, Macklin, Shanghai, China), poly(vinylidene fluoride-hexafluoro propylene) (PVDF-HFP, Mn = 600,000, Arkema, Colombes, France), 2hydroxy-2-methyl-1-phenylpropane-1-one (HMPP, RYOJI, Frankfurt, Germany), polyurethane acrylate (PUA, RYOJI, Frankfurt, Germany), Li_1.3_Al_0.3_Ti_1.7_(PO_4_)_3_ (LATP, 99.99% purity, Macklin), N,N-dimethylformamide (DMF, Macklin), and Li_4_Ti_5_O_12_ (LTO, Macklin, Shanghai, China) powder.

### 2.2. Preparation of LTO Electrodes

**Figure 1:**
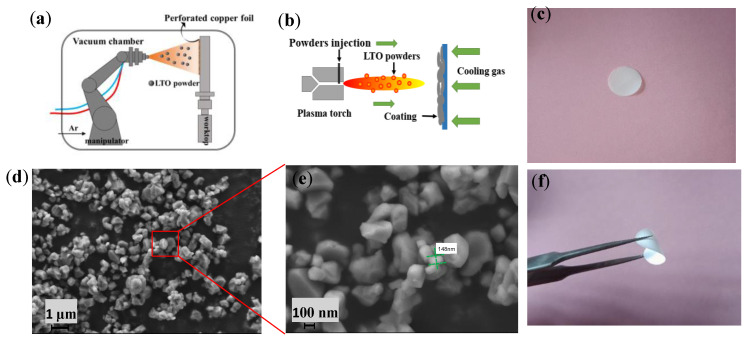
(**a**,**b**) Schematic diagram of LTO electrode preparation, (**d**,**e**) LTO particle map, (**c**,**f**) macroscopic diagram of solid electrolyte membrane.

LTO powder (Figure 1d,e) was sprayed onto the perforated copper foil at high speed using plasma spraying technology. The perforated copper foil with a thickness of 15 μm was fixed on a copper plate with good heat dissipation. The main gas in the plasma was argon, and hydrogen was used as an auxiliary gas. Spraying parameters are shown in Table 1. The spraying distance was 100 mm, and the plasma current was varied between 500 and 600 A. The macroscopic view of the LTO electrode with a schematic diagram of the preparation process is shown in Figure 1.

### 2.3. Preparation of Composite Solid State Electrolyte

**Figure 2:**
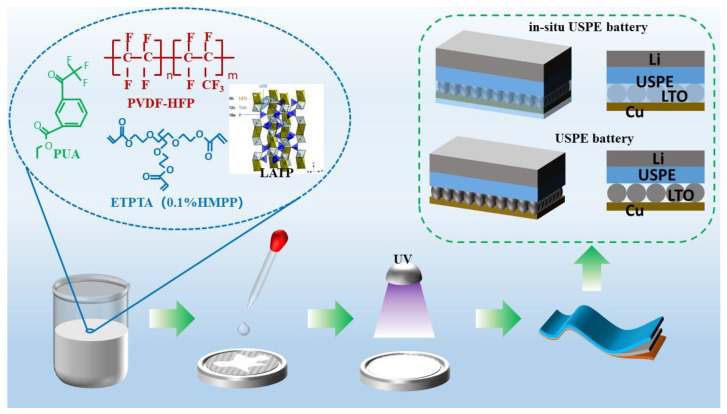
Schematic diagram of the preparation process of in situ polymer-ceramic composite solid-state electrolyte.

The composite solid electrolyte was prepared by UV solidification method, and the preparation process is shown in Figure 2. 2 g of PVDF-HFP was dissolved in 8 g of DMF, and after dissolving into a transparent viscous solution with stirring at 45 °C, 0.2 g of LATP was added to continue heating and stirring for 6 h. After stirring, 0.5 g of PUA and 0.8 g of ETPTA and HMPP (the concentration of HMPP was fixed at 0.1 wt% of ETPTA) were added. Stirring was continued for 5 min, and the obtained electrolyte precursor solution was poured onto the electrode sheet in a small amount uniformly. The electrolyte precursor solution was uniformly applied to the electrode sheet with a 150 µm spatula, and the remaining electrolyte precursor solution was poured onto the polytetrafluoroethylene (PTFE) plate, after which the electrolyte-stained electrode sheet and the PTFE plate with precursor solution were put into a UV curing machine for 5 min of light solidification and then placed into a drying oven at 60 °C for 30 min. The dried composite electrode sheet and solid electrolyte were cut into Φ18 discs; the solid electrolyte was named USPE as the control.

### 2.4. LTO Battery Assembly

CR2025-type button cell batteries were assembled in a glove box filled with argon gas. The in situ electrode sheet was immersed in liquid electrolyte for 5 min before battery assembly, and the in situ electrolyte plasma-sprayed LTO electrode sheet was used as the cathode, and lithium metal was used as the anode. The battery was assembled according to the operational steps.

### 2.5. Characterization and Electrochemical Measurements

The physical phase of the samples was analyzed by X-ray diffraction (XRD, D8-ADVANCE, Bruker, Karlsruhe, Germany, Cu, 40 k V, 40 m A) with a radiation angle between 10° and 90° in steps of 0.1°. A scanning electron microscope (SEM; GeminiSEM 500, ZEISS Inc., Jena, Germany) was used to observe the microscopic morphology of the plasma-sprayed LTO electrodes. The prepared in situ battery samples were charged and discharged using the NEWARE battery test system (CT-4008). Electrochemical impedance spectroscopy (EIS) was performed on the samples using an electrochemical workstation (CHI660E) in the frequency range of 0.1~10^5^ Hz at room temperature.

### 2.6. Electrochemical Measurements

The timing current of the lithium symmetric battery was tested at a voltage of 0.5 mV, lasting 4000 s, and the formula was as follows:

(1) $$t_{{Li}^{+}}=\frac{I_{s}(∆V-I_{0}R_{0})}{I_{0}(∆V-I_{s}R_{s})}$$

The lithium-ion transference number (*t_Li_*_+_) can be obtained. *I*_0_ and *I_s_* are current values after DC polarization starts and stabilizes, *R*_0_ and *R*_s_ are the impedance values before and after the DC polarization, and ∆V is the value of the voltage applied to both ends of the battery. For the ionic conductivity test, the battery assembly used stainless steel (SS) as a symmetrical battery and an electrochemical impedance test together to calculate the ionic conductivity. The frequency range of the impedance test is 0.1~10^6^ Hz.

(2) $$\sigma =\frac{L}{S\times R}$$

where σ represents the ionic conductivity, *L* is the thickness of the electrolyte, *S* represents the contact area between the electrolyte and the test electrode (SS), and *R* is the impedance value of the battery electrolyte measured by EIS. Linear sweep voltammetry (LSV) was used to perform electrochemical window tests at 2.5~6 V at a scanning rate of 0.1 mV s^−1^.

## 3. Results

**Figure 3:**
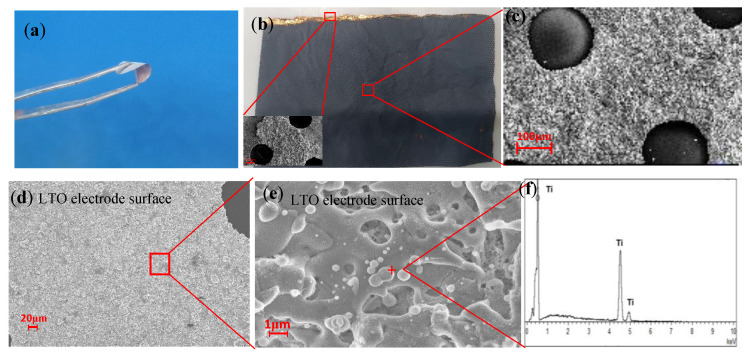
(**a**,**b**) Image of an LTO electrode prepared by atmospheric plasma spraying. (**c**–**e**) SEM images of LTO electrodes. (**f**) LTO electrode EDS.

We measured the active material loading of the LTO electrode monolith as 3.8 mg and the total mass loading as 5.4 mg. As shown in Figure 3, after plasma spraying, the SEM image of the LTO electrode shows that a lithium titanate functional coating was formed by stacking on the porous copper foil, with a small amount of unmelted spherical, LTO small particles on the surface. Figure 3a shows a macro image of the LTO electrodes and in situ solid electrolyte, which indicates that both LTO electrodes and solid electrolytes are flexible. Figure 3b shows the macroscopic view of the LTO electrode after spraying, indicating that the battery electrode made in this way does not need the complex process of slurry coating, and the electrode size is no longer limited by the limitations of the scraping and coating tools, which can meet the needs of personalization. Figure 3c,d show the microscopic magnification of the macroscopic electrode; it can be observed that the LTO powder is uniformly attached to the porous copper foil. Figure 3e is a further enlarged micrograph of the micro surface of the LTO electrode, from which the stacked morphology of the electrode surface can be observed, indicating that during the spraying process, the LTO powder is melted and stacked onto the porous copper foil and then cooled to form the stacked morphology. A large number of Ti and O are observed on the LTO electrode surface (Figure 3f).

**Figure 4:**
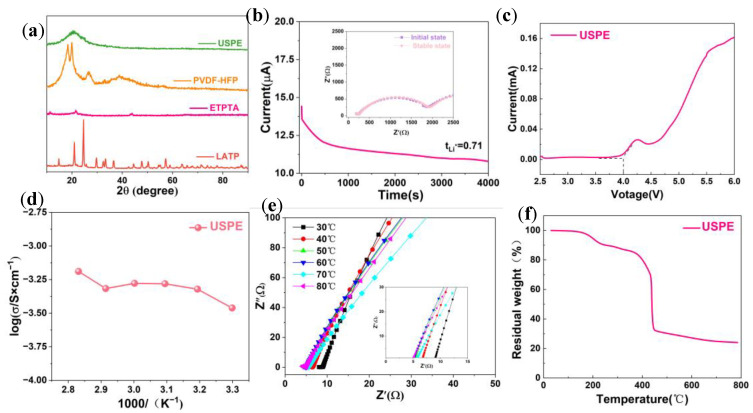
(**a**) XRD for USPE, PVDF-HFP, LATP, and ETPTA. (**b**) Room temperature chronoamperometry of the Li/USPE/Li cell at a potential step of 5 mV and AC impedance spectra of the symmetric cell before and after polarization (inset). (**c**) LSV curves of USPE at a scanning rate of 0.1 mV s^−1^ at 25 °C. (**d**) Arrhenius plots for the ionic conductivity of USPE at different temperatures. (**e**) Nyquist curves of USPE in the frequency range of 0.1–10^6^ MHz in the temperature range of 30° to 80 °C. (**f**) TG diagram of USPE.

The phase structure of the polymer electrolyte was investigated by XRD (Figure 4a). Pure ETPTA showed three visible crystal diffraction peaks near 12.8°, 22.3°, and 45.8°. The composite solid electrolyte USPE showed broad peaks between 15° and 25° and no other crystallization peaks, indicating that the USPE was in an amorphous state, which is conducive to the enhancement of Li^+^ ion transfer in the composite electrolyte [28]. ETPTA monomers with three C=C double bonds act as cross-linking agents and can form three-dimensional cross-linked networks by free radical polymerization. The ETPTA crosslinked network and PVDF-HFP chains can form a semi-interpenetrating network skeleton, in which the linear PUA chains and PVDF-HFP chains can soften the ETPTA rigid network, thus improving the contact between the electrodes and the electrolyte [29].

The lithium ion transference number (*t_Li_*_+_) before and after DC polarization was determined by combining the timed-current method and electrochemical impedance spectroscopy (EIS) measurements, as shown in Figure 4b.

According to the BVE equation [19], *t_Li_*_+_ = 0.71 for the USPE electrolyte at room temperature. Figure 4c shows that the electrochemical window is higher than 4.0 V, indicating that the USPE electrolyte has high voltage resistance characteristics. As shown in Figure 4d, the ionic conductivity of USPE exhibits an increasing trend with increasing temperature, and the ionic conductivity reaches a maximum value of 0.65 mS cm^−1^ at 80 °C. Not only that, USPE also exhibits good ionic conductivity of up to 0.35 mS cm^−1^ at 30 °C. Figure 4e shows that the impedance values of the USPE electrolyte at different temperatures display a minimum impedance at 80 °C. Figure 4f shows that USPE has two weight loss stages. The first stage occurs at 190 °C due to the evaporation of a small amount of water remaining in USPE. The second stage occurs at 400 °C, and the reason for weight loss is thermal reactions, indicating that the USPE electrolyte has high thermal stability [30].

**Figure 5:**
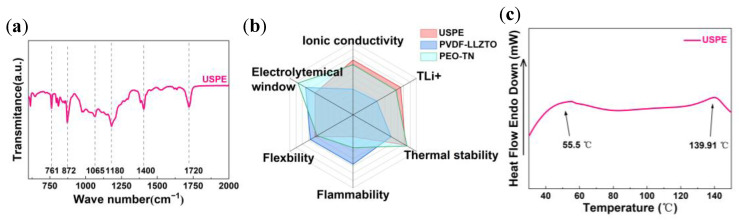
(**a**) FTIR spectra of USPE membranes. (**b**) Radar plots that show performance of USPE, PVDF-LLZTO [32], and PEO-TN [33]. (**c**) DSC curves of USPE membranes.

Figure 5a depicts the FITR spectra of the USPE electrolyte. The characteristic peaks appearing at 761, 872, 1065, and 1400 cm^−1^ represent the CF_2_ bending and skeletal bending, C-H rocking, amorphous phase belt, C-C stretching, and CH_2_ deformation, which come from pure PVDF-HFP [21]. Figure 5c depicts the DSC spectra of USPE. The pure PVDF-HFP membrane has an endothermic peak near 146.1 °C, which is the melting temperature (T_m_) of PVDF-HFP [22]. Obviously, an endothermic peak appears in the DSC curve near 55 °C, indicating that the Tm of ETPTA and PVDF-HFP decreased to 55.5 °C. It is worth mentioning that the lower crystallinity is beneficial to increasing the disordered region of the system, which makes the organic chain more disordered and free and enhances the ionic conductivity and lithium ion migration number of the electrolyte. The membrane has the endothermic peak at around 146.1 °C, which is the melt temperature (Tm) of the PVDF-HFP [31]. Figure 5b shows the performance radar diagrams of the three electrolytes of USPE, PVDF-LLZTO [32], and PEO-TN [33]. USPE showed good thermal stability, high ionic conductivity, and high *t_Li_*_+_. The ionic conductivity and *t_Li_*_+_ of USPE are greater than those of the other two electrolytes. The ionic conductivity and *t_Li_*_+_ are important factors to evaluate the solid electrolyte. A high ionic conductivity and *t_Li_*_+_ can reduce the concentration polarization of the battery during charging and discharging, showing a better rate performance and smaller resistance. However, the electrochemical window of USPE is smaller than that of the other two electrolytes, but because the operating voltage (1–3 V) of LTO is much smaller than 4 V, the lower electrochemical window has little effect on the LTO battery. Overall, the USPE electrolyte exhibits good overall performance in terms of ion conductivity, *t_Li_*_+_, electrochemical window, good flexibility, thermal stability, and flammability. Overall, the USPE electrolyte exhibited good overall performance in terms of ion conductivity, *t_Li_*_+_, electrochemical window, good flexibility, thermal stability, and flammability.

**Figure 6:**
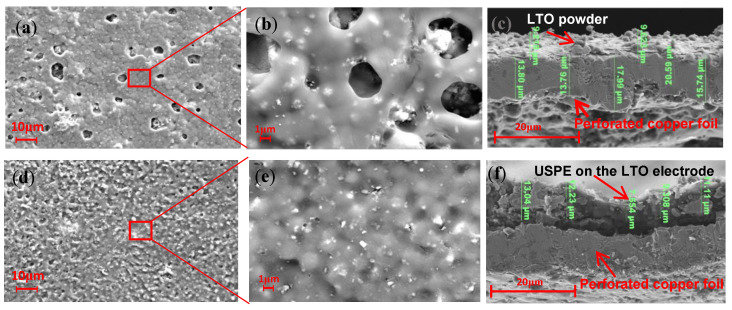
SEM images of (**a**,**b**) surface of USPE on the LTO electrodes. (**c**) LTO electrode cross-section. (**d**,**e**) Surface of USPE on the LTO electrodes after 600 cycles. (**f**) Cross-section of in situ USPE on the LTO electrode.

As shown in Figure 6a,b, the surface of the in situ USPE electrolyte sheet before cycling is covered with several small pores of 10 µm or less, and after magnification to 5000×, as shown in Figure 6b, the white dots on the surface of the in situ USPE electrolyte are the Li_1.3_Al_0.3_Ti_1.7_(PO_4_)_3_ (LATP) ceramic particles, and the LATP ceramic particles can be seen to be uniformly distributed on the electrode surface. As shown in Figure 6e, the morphology of the LATP ceramic particles changes compared with the surface of the electrolyte before the cycle. The well-crystallized original LATP particles before the cycle are broken into smaller particles, which means that the LATP in the composite electrolyte reacts with the metal lithium, and the lithiation product is mainly the lithium-rich phase Li_3_Al_0.3_Ti_1.7_ (PO_4_)_3_. Therefore, it can be inferred that the white particles in Figure 6e should be the lithium-rich phase Li_3_Al_0.3_Ti_1.7_ (PO_4_)_3_. This lithiation product has a high electronic conductivity, resulting in a series of chemical reactions and destroying the interface between the electrolyte and the electrode [34]. From the plasma-sprayed electrode cross-section SEM image (Figure 6c), it can be seen that the thickness of the sprayed LTO is about 2–5 µm. The thickness of the USPE electrolyte can be seen in the in situ electrode cross-section SEM image, which is about 7–14 µm (Figure 6f).

**Figure 7:**
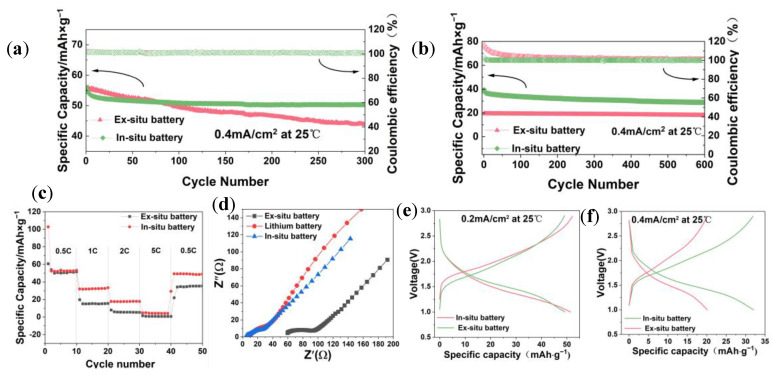
(**a**) Cyclic test performance profiles of (**a**,**b**) in situ cell and ex situ cell at 0.5 C and 1 C under room temperature. (**c**) Rate performance of the cells with two batteries. (**d**) EIS of in situ, ex situ, and liquid batteries. (**e**,**f**) Charge/discharge curves for in situ and ex situ batteries.

Figure 7 shows the charge/discharge, cyclic, and rate performance of the in situ and ex situ batteries. Figure 7a shows that the first stabilized discharge specific capacity of the in situ battery reached 56 mAh g^−1^ at 0.2 mA/cm^2^, the capacity retention of the in situ battery is 90%, and the capacity retention of the ex situ battery is 78.8% after 300 cycles. Furthermore, after 600 cycles at 0.4 mA/cm^2^, the first discharge specific capacity of the in situ battery reached 39.4 mAh g^−1^, close to two times the discharge specific capacity (19.75 mAh g^−1^) of the ex situ battery (Figure 7b). The capacity retention rate of the in situ battery was 76.4%, and the capacity retention rate of the ex situ battery was about 89.8%. It indicates that in the case of increasing current density, the capacity fading of in situ batteries is smaller, and they have a long cycle life. The rate performance curve (Figure 7c) shows that the in situ battery can maintain a discharge specific capacity of approximately 22 mAh g^−1^ at a current density of 0.8 mA/cm^2^, while the ex situ battery specific capacity decreases to 0 mAh g^−1^, indicating that the in situ battery can withstand higher charge/discharge rates. This is because in situ batteries avoid the large interface impedance generated by point contacts between electrodes and solid electrolytes (Figure 7d),and are more adaptable to volume changes during battery charging and discharging processes. Figure 7e,f show the charge/discharge curves of in situ and ex situ batteries at 0.4 mA/cm^2^ and 0.8 mA/cm^2^. The voltage plateaus of the two batteries are close to each other, and the charge/discharge specific capacities of both in situ batteries are greater than those of the ex situ batteries. Overall, the electrochemical performance of in situ batteries is superior to that of ex situ batteries.

## 4. Conclusions

In this study, amorphous LTO electrodes were prepared by the plasma spraying method, composite electrolytes were prepared using the UV curing method, and the electrochemical performance of the in situ and ex situ batteries were comparatively investigated. The conclusions are as follows:

(1) The LTO electrode was prepared by the atmospheric plasma method, and both the LTO electrode and the solid electrolyte were flexible and bendable. After being assembled into an LTO//electrolyte//Li battery, it exhibited excellent rate and cycle performance, which proves that the preparation method is practically feasible.

(2) The UV curing process is an efficient method for preparing PVDF HFP/LATP composite solid electrolytes, which have a high ion conductivity (0.35 mS cm^−1^ at 30 °C) and electrochemical window (above 4.0 V).

(3) Compared with the LTO//ex situ electrolyte//Li solid-state battery, the capacity retention rate of the LTO//in situ electrolyte//Li solid-state battery was 90% after 300 cycles at 0.2 mA/cm^2^, and the capacity retention rate was 76.4% after 600 cycles at 0.4 mA/cm^2^.

This work provides a new strategy and process method for the design and large-scale manufacturing of large-area, long-life lithium solid-state batteries.

## Figures and Tables

**Table 1:** Spray data table.

Coating Material	Current/A	Ar/L min^−1^	H_2_/L min^−1^	Spray Gun Moving/mm s^−1^	Spray Distance/mm
LTO	500–600	40	11	1000	100

## Data Availability

Data are contained within the article.
